# Registration, results reporting, and publication bias of clinical trials supporting FDA approval of neuropsychiatric drugs before and after FDAAA: a retrospective cohort study

**DOI:** 10.1186/s13063-018-2957-0

**Published:** 2018-10-23

**Authors:** Constance X. Zou, Jessica E. Becker, Adam T. Phillips, James M. Garritano, Harlan M. Krumholz, Jennifer E. Miller, Joseph S. Ross

**Affiliations:** 1 Yale School of Medicine, New Haven, CT USA; 2 Department of Psychiatry, Massachusetts General Hospital, Boston, MA USA; 3 McLean Hospital, Boston, MA USA; 4 Harvard Medical School, Boston, MA USA; 5 Division of Cardiovascular Medicine, Department of Internal Medicine, Beth Israel Deaconess Medical Center, Boston, MA USA; 6 Section of Cardiovascular Medicine, Department of Internal Medicine, Yale School of Medicine, New Haven, CT USA; 7 Department of Health Policy and Management, Yale School of Public Health, New Haven, CT USA; 8 Center for Outcomes Research and Evaluation, Yale-New Haven Hospital, New Haven, CT USA; 9 Section of General Medicine, Department of Internal Medicine, Yale School of Medicine, PO Box 208093, New Haven, CT 06520 USA

**Keywords:** FDAAA, ClinicalTrials.gov, Clinical trial registration, Clinical trial results, Selective publication, Publication bias

## Abstract

**Background:**

Mandatory trial registration, and later results reporting, were proposed to mitigate selective clinical trial publication and outcome reporting. The Food and Drug Administration (FDA) Amendments Act (FDAAA) was enacted by Congress on September 27, 2007, requiring the registration of all non-phase I clinical trials involving FDA-regulated medical interventions and results reporting for approved drugs. The association between FDAAA enactment and the registration, results reporting, and publication bias of neuropsychiatric trials has not been studied.

**Methods:**

We conducted a retrospective cohort study of all efficacy trials supporting FDA new drug approvals between 2005 to 2014 for neuropsychiatric indications. Trials were categorized as pre- or post-FDAAA based on initiation and/or completion dates. The main outcomes were the proportions of trials registered and reporting results in ClinicalTrials.gov, and the degree of publication bias, estimated using the relative risks pre- and post-FDAAA of both the publication of positive vs non-positive trials, as well as of publication of positive vs non-positive trials without misleading interpretations. Registration and results reporting proportions were compared pre- and post-FDAAA using the two-tailed Fisher exact test, and the degrees of publication bias were compared by calculating the ratio of relative risks (RRR) for each period.

**Results:**

The FDA approved 37 new drugs for neuropsychiatric indications between 2005 and 2014 on the basis of 142 efficacy trials, of which 101 were pre-FDAAA and 41 post-FDAAA. Post-FDAAA trials were significantly more likely to be registered (100% vs 64%; *p* < 0.001) and report results (100% vs 10%; *p* < 0.001) than pre-FDAAA trials. Pre-FDAAA, positive trials were more likely to be published (relative risk [RR] = 1.52; 95% confidence interval [CI] = 1.17–1.99; *p* = 0.002) and published without misleading interpretations (RR = 2.47; CI = 1.57–3.73; *p* < 0.001) than those with non-positive results. In contrast, post-FDAAA positive trials were equally likely to have been published (RR = 1; CI = 1–1, *p* = NA) and published without misleading interpretations (RR = 1.20; CI = 0.84–1.72; *p* = 0.30). The likelihood of publication bias pre-FDAAA vs post-FDAAA was greater for positive vs non-positive trials (RRR = 1.52; CI = 1.16–1.99; *p* = 0.002) and for publication without misleading interpretations (RRR = 2.06, CI = 1.17–3.61, *p* = 0.01).

**Conclusions:**

The enactment of FDAAA was followed by significantly higher proportions of trials that were registered and reporting results on ClinicalTrials.gov and significantly lower degrees of publication bias among trials supporting recent FDA approval of drugs for neuropsychiatric indications.

**Electronic supplementary material:**

The online version of this article (10.1186/s13063-018-2957-0) contains supplementary material, which is available to authorized users.

## Background

Research suggests that as many as 15–50% of completed clinical studies are never published [1–10] and that publication bias persists: trials with non-positive results are significantly more likely to remain unpublished than trials with positive results [3, 11–15]. Although under- or non-publication of clinical trials is a problem ubiquitous in medicine and science, many considered the problems to be particularly severe among clinical studies in neurology and psychiatry [16]. Clinical studies supporting approved drugs for neuropsychiatric indications, such as paroxetine (Paxil) [17], reboxetine (Edronax) [18], gabapentin (Neurontin) [19], and lamotrigine (Lamictal) [20], have been identified as being subject to underreporting. Data demonstrating these drugs to be potentially ineffective for approved indications or suggesting harm were not publicly disclosed until the pharmaceutical companies’ internal documents were reviewed during legal proceedings [16, 21, 22]. Similarly, among trials evaluating drugs indicated for depression [7], anxiety [23], and psychotic disorders [24] that were first approved by the US Food and Drug Administration (FDA) in recent decades, 80–90% of trials with negative or equivocal results were either not published or were published in a misleading manner to suggest a positive result, while nearly 100% of trials with positive results were published.

A publicly accessible, centralized trial registry can prevent clinical trials from remaining hidden by allowing interested individuals, including patients, clinicians, and regulators, to identify all trials relevant to a specific medical condition or to a specific medical product. In 1997, Congress passed the FDA Modernization Act (FDAMA), which mandated the first USA-based public registry ClinicalTrials.gov [25] in 2000, maintained by the National Institutes of Health (NIH) [26]. In 2005, the International Committee of Medical Journal Editors (ICMJE) issued a policy requiring trial registration as a condition of publication in member journals [27], which was followed by an increase in registered trials [28–31]. Nonetheless, the FDAMA only required registration of a small number of trials, while the ICMJE recommendation was only followed on a voluntary basis and still permitted publication of unregistered trials [32, 33].

In 2007, Congress passed the FDA Amendments Act (FDAAA) [34], applicable to essentially all non-phase I interventional studies involving FDA-regulated drugs, biological products, or devices. The FDAAA mandated that sponsors and investigators register all such trials in ClinicalTrials.gov prior to subject enrollment and report results to ClinicalTrials.gov within 30 days post approval of the indication being studied. The FDAAA is applicable to trials that began after September 27, 2007 and to earlier trials that were still ongoing as of December 26, 2007. Inappropriately delayed registration and results reporting, as well as reporting of false results, are punishable by fines of up to $10,000 per day and can lead to withholding of funding from studies receiving federal support.

It has now been ten years since the FDAAA was enacted. Its impact on clinical trial registration, results reporting, and publication bias has largely remained undetermined. One recent study demonstrated that the FDAAA was associated with increased registration and publication of clinical studies of new drugs approved to treat cardiovascular disease and diabetes [35]. However, no study has focused on trials involving drugs treating neurological and psychiatric conditions, an area in which concern for selective publication and outcome reporting remains.

The objective of our study was to compare the proportions of trials that were registered and reporting results on ClinicalTrials.gov, as well as the degree of publication bias, before and after the FDAAA, among clinical trials supporting FDA approval of drugs for neuropsychiatric indications. We designed a retrospective cohort study using a cohort of clinical trials that were submitted to and reviewed by the FDA for the approval of New Drug Applications (NDAs) between 2005 and 2014 for the treatment of neurologic and psychiatric conditions. For each trial, we determined their FDAAA eligibility based on trial initiation and/or completion dates [34], as well as their registration, results reporting, and publication status.

## Methods

### Data sources

Data were obtained from three sources: Drugs@FDA [36], ClinicalTrials.gov, and PubMed’s listing of MEDLINE-indexed journals. Drugs@FDA is a public database maintained by the FDA, providing access to regulatory actions and documents issued for each drug approved by the agency. ClinicalTrials.gov is a public clinical trial registry database maintained by the National Library of Medicine at the NIH [25]. PubMed’s list of MEDLINE-indexed journals includes more than 5500 biomedical journals.

### Novel therapeutics approved for treating neurological and psychiatric disorders, 2005–2014

The Center of Drug Evaluation and Research, which is part of the FDA, provides annual reports summarizing all NDAs approved in each year [36, 37]. We downloaded the reports from 2005 to 2014, when available, and otherwise searched Drugs@FDA for those NDAs that were approved to treat neurologic and psychiatric disorders. Our study sample began with drugs approved in 2005 to align with our prior work [38] and because an earlier seminal study on the topic examined all antidepressants approved through 2004 [7]; we chose to exclude drugs approved after December 2014 to ensure that at least 24 months had passed between drug approval date and the date when we concluded the final search for the registration record, reported results, and publication, which was March 2017. For each NDA, we recorded its indication, orphan status, priority review status, accelerated approval status, sponsor, and approval date.

### Efficacy trials supporting FDA new neuropsychiatric drug approval

As described in a comprehensive tutorial for how to use Drugs@FDA [39], we downloaded the relevant FDA files for each NDA from Drugs@FDA, including the approval letters, summary reviews, clinical reviews, and statistical reviews. Among these files, we searched for clinical trials evaluating the efficacy of the drugs under review. We included only trials for which the FDA discussed and characterized results, based on the assumption that these trials influenced the FDA’s decision to approve the study drug for the proposed indication. We excluded ongoing trials, phase I/safety-only trials, expanded access trials, terminated and withdrawn trials without enrollment, and trials evaluating indications different than that for which the drugs were originally approved. We also excluded failed trials. For each included trial, we recorded the following characteristics: pivotal status, phases, sponsors, study sites, trial length, randomization, blinding, types of control, description of the treatments, arms of the investigational drugs, enrollment numbers, and the primary efficacy endpoints. A pivotal study is defined by the FDA as “a definitive study in which evidence is gathered to support the safety and effectiveness evaluation of the medical product for its intended use” [40]. Pivotal status was frequently assigned prospectively by the FDA, occasionally assigned retrospectively by the FDA, or at times not assigned by the FDA and thus determined using a previously described method [38].

### Determination of FDAAA status

The FDAAA, as enacted in 2007, clarified that new requirements would apply to trials that were initiated after September 27, 2007, as well as to trials initiated earlier but still ongoing as of December 26, 2007. Based on this, FDAAA applicable trials were categorized as post-FDAAA, while trials that were initiated or completed prior to the cut-off dates were categorized as pre-FDAAA.

### Determination of registration and results reporting status on ClinicalTrials.gov

To determine whether trials were registered and reported results on ClinicalTrials.gov, one investigator (CXZ) performed the initial search using the following terms and their combinations: generic, or brand names of the study drugs, drug indications, trial IDs, trial acronyms, numbers of participants randomized, comparators, and study time frames. For trials that were not able to be matched with any registration record, a second investigator (JEB) independently performed a second round of searches. No new records were identified.

### Determination of publication status

To determine whether trials were published, we searched PubMed for full-length publications using the same terms as we did for the registration record. Among identified publications, abstracts and conference reports were excluded. Publications reporting multiple trials, such as reviews and meta-analyses, were also excluded unless the results of each trial were analyzed and discussed individually in the level of detail as one would expect from a full-length publication. When the search terms returned too many similar entries in PubMed, we used Google Scholar to narrow the results. Google Scholar has the advantage that it can search among the full texts of publications hosted by a variety of online databases or platforms, while for many journals, especially those that require paid access, PubMed searches only among the titles and abstracts. We provide more detail on our search strategy in Additional file 1.

### Interpretation of trial results: publication vs FDA

Trials were classified as positive, negative, or equivocal based on the FDA’s interpretation of the results as described in Additional file 1. The classification was based on whether the primary outcome(s) achieved statistical significance while taking into consideration the summary statements made by the FDA medical reviewers regarding whether or not the findings provided support for the efficacy claim of the study drugs. Published trial results were categorized similarly based on whether the primary outcomes achieved statistical significance according to the authors’ analysis while taking into consideration the authors’ conclusions in the abstract section. Trials with equivocal or negative results were grouped together as non-positive trials for purposes of calculating publication bias.

### Validating the published interpretations

We validated the interpretations of the trial results made by the study investigators for each publication using the interpretations made by the FDA medical reviewers found in the FDA approval package as the gold standard. Both the conclusions in the abstract and the main text of the publications were validated. The two were considered in agreement if the interpretations were both categorized as positive, negative, or equivocal, and no major contradictions existed between the two statements. As an example of contradiction between two sources: the published interpretation of trial 02 of milnacipran (Savella) concluded that “both doses (100 and 200 mg/d) were associated with significant improvements in pain and other symptoms” [41]. This was considered different from the statement made by the FDA in the summary review documents, which stated that “[the] analysis of the ‘pain only’ responders does not indicate that there is a significant effect of MLN (Savella) on pain….(treatment effect) was driven by the patient global response outcome rather than the pain or function outcome…when studied in isolation, statistically significant treatment effects for pain and function were not demonstrated” [42]. Due to the interpretive nature of this comparison, two additional investigators (JEB and JSR) reviewed all instances where there was disagreement between the FDA’s and the publications interpretation.

### Calculating the degree of publication bias

We calculated and compared two different measures of publication bias between pre- and post-FDAAA trials. First, we estimated the relative risk of publishing positive vs non-positive trials in each period. Second, we estimated the relative risk of publishing positive vs non-positive trials without misleading interpretations in each period. Thus, publication bias was calculated as the ratio of relative risks (RRR) pre-FDAAA vs post-FDAAA.

### Data validation

Registration status, results reporting status, publication status, and publication-FDA interpretation agreement were validated as described previously. We performed quality control and data validation, having a second investiator (JEB) re-collect all data but not reported for purposes of this study. A second investigator (JEB) re-collected all data elements obtained for a random 10% sample of the included new drug approvals, using an online randomization tool [43] to randomly select 4 out of the 37 drugs. Among the 676 unique data elements collected by the two investigators, the rate of agreement was 99.6%, and disagreements were resolved through consensus.

### Data analysis

We used descriptive statistics to characterize the proportions of trials that were registered and reporting results on ClinicalTrials.gov. We used two-tailed Fisher exact tests to compare the proportions among pre- and post-FDAAA trials. Analysis was performed using Epi Info Companion App for iOS version 3.1.1 (Centers for Disease Control and Prevention [CDC]; Atlanta, GA) [44], as well as MedCalc online statistical software [45], supplemented using an online program written by Hutchon [46] to calculate the RRRs to estimate both measures of publication bias.

## Results

### Characteristics of the neuropsychiatric drugs approved between 2005 and 2014

Between January 1, 2005 and December 31, 2014, 37 new drugs were approved by the FDA for the treatment of neuropsychiatric conditions, of which 23 (62%) were approved for neurological conditions and 14 (38%) for psychiatric disorders, which included 3 drugs for substance-use related conditions (Table 1). Among the 37 approved drugs, 34 (92%) were pharmacologic therapies, 3 (8%) were biologics; orphan status was granted for 9 (24%), priority review status for 6 (17%), and accelerated approval for 1 (3%) (see Additional file 2).

**Table 1:** New Drug Applications (NDAs) Approved by the FDA between 2005 and 2014 with indications for neurologic and psychiatric conditions

Brand Name	INN Name	NDA applicant	Indication	Approval year
Rozerem	Ramelteon	Takeda Global	Insomnia	2005
Chantix	Varenicline tartrate	Pfizer	Smoking cessation	2006
Azilect	Rasagiline mesylate	Teva	Parkinson’s disease	2006
Invega	Paliperidone	Janssen, L.P.	Schizophrenia	2006
Vyvanse	Lisdexamfetamine dimesylate	New River	Attention-deficit hyperactivity disorder	2007
Neupro	Rotigotine	Schwarz Bioscience	Parkinson’s disease	2007
Pristiq	Desvenlafaxine succinate	Wyeth	Major depressive disorder	2008
Relistor	Methylnaltrexone bromide	Progenics	Opioid-induced constipation	2008
Xenazine	Tetrabenazine	Prestwick	Huntington’s disease	2008
Vimpat	Lacosamide	Schwarz Bioscience	Partial-onset seizure disorder	2008
Banzel	Rufinamide	Eisai Inc.	Seizures associated with Lennox-Gastaut syndrome	2008
Nucynta	Tapentadol Hydrochloride	Ortho-McNeil-Janssen	Acute pain	2008
Lusedra	Fospropofol disodium	Eisai Medical	Anesthesia	2008
Savella	Milnacipran yydrochloride	Cypress Bioscience Inc.	Fibromyalgia	2009
Dysport	AbobotulinumtoxinA	Ipsen Biopharm Limited	Cervical dystonia	2009
Fanapt	Iloperidone	Vanda Pharmaceuticals Inc	Schizophrenia	2009
Saphris	Asenapine maleate	Organon USA Inc.	Bipolar I disorder	2009
Sabril	Vigabatrin	Lundbeck Inc.	Complex partial seizure disorder	2009
Qutenza	Capsaicin	NeurogesX Inc.	Neuropathic pain	2009
Ampyra	Dalfampridine	Acorda Therapeutics Inc.	Multiple sclerosis	2010
Xeomin	IncobotulinumtoxinA	Merz Pharmaceuticals GmbH	Cervical dystonia and blepharospasm	2010
Gilenya	Fingolimod	Novartis Pharmaceuticals Corp.	Multiple sclerosis	2010
Latuda	Lurasidone hydrochloride	Sunovion Pharmaceuticals Inc.	Schizophrenia	2010
Viibryd	Vilazodone hydrochloride	Trovis Pharmaceuticals LLC	Major depressive disorder	2011
Horizant	Gabapentin enacarbil	Glaxo Group Ltd. DBA GlaxoSmithKline	Restless legs syndrome	2011
Potiga	Ezogabine	GlaxoSmithKline	Partial seizure disorder	2011
Onfi	Clobazam	Lundbeck Inc.	Seizures associated with Lennox-Gastaut syndrome	2011
Aubagio	Teriflunomide	Sanofi Aventis US LLC	Multiple sclerosis	2012
Fycompa	Perampanel	Eisai Inc.	Partial seizure disorder	2012
Tecfidera	Dimethyl fumarate	Biogen Idec INC	Multiple sclerosis	2013
Trintellix (formerly Brintellix)	Vortioxetine	Takeda Pharmaceuticals USA Inc.	Major depressive disorder	2013
Aptiom	Eslicarbazepine acetate	Sunovion Pharmaceuticals Inc.	Partial seizure disorder	2013
Hetlioz	Tasimelteon	Vanda Pharmaceuticals Inc.	Non-24 h sleep-wake disorder	2014
Northera	Droxidopa	Chelsea Therapeutics Inc.	Neurogenic orthostatic hypotension	2014
Belsomra	Suvorexant MK4305	Merck Sharp & Dohme Corp.	Insomnia	2014
Plegridy	Peginterferon beta-1A	Biogen Idec Inc.	Multiple sclerosis	2014
Movantik	Naloxegol	AstraZeneca Pharmaceuticals LP	Opiod-induced constipation	2014

*INN* international nonproprietary name

Please refer to the drug label for full description of each drug indication

### Clinical trials supporting FDA approval

**Fig. 1:**
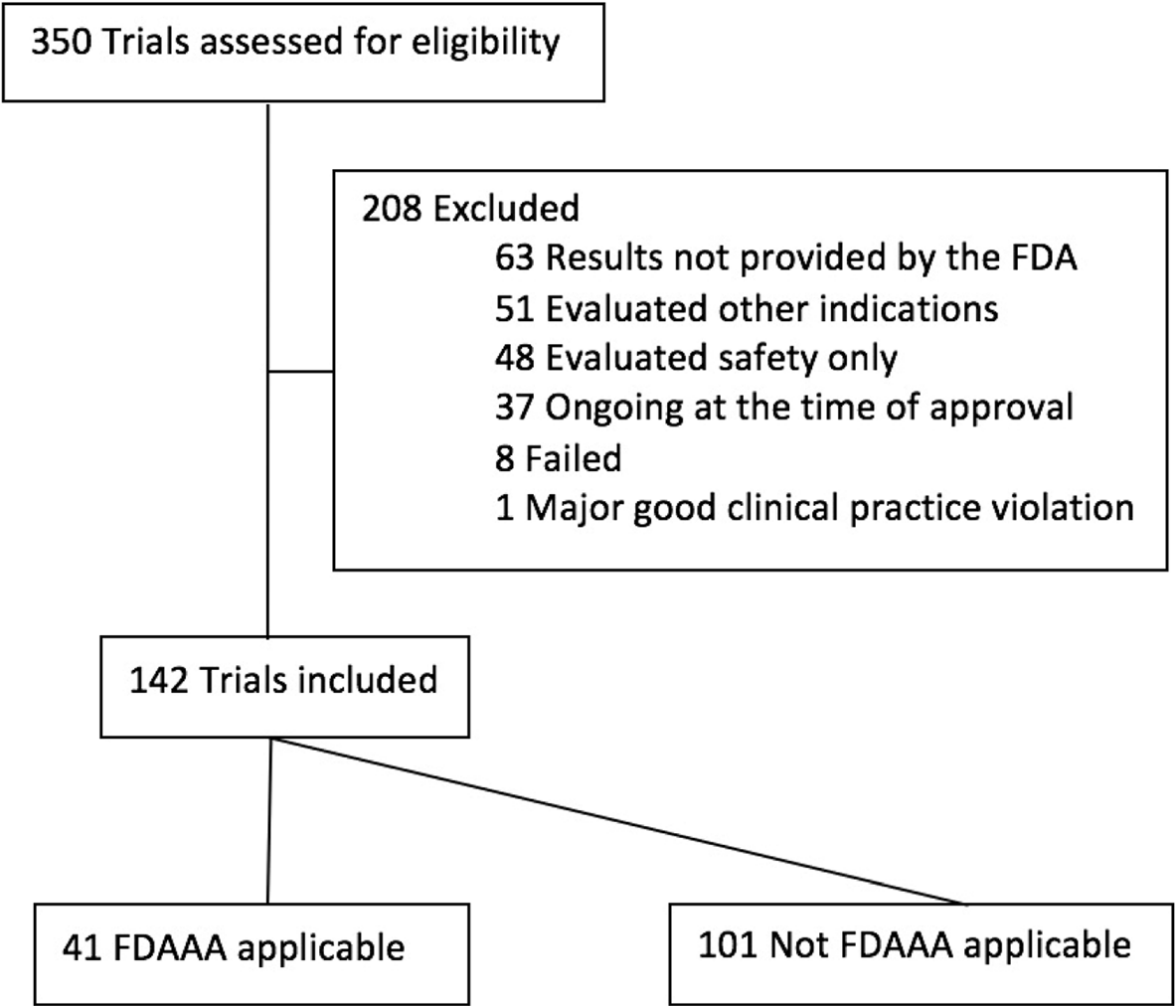
Identification of trials reviewed by the FDA for New Drug Applications with neuropsychiatric indications, 2005–2014. Flow chart depicting the process of selecting efficacy trials conducted to support new neuropsychiatric drugs and reviewed by the FDA for drug approval between 2005 and 2014. Trials can be excluded for satisfying one or more exclusion criteria

There were 142 trials that supported the approval of these 37 neuropsychiatric drugs (Fig. 1), of which 101 (71%) were categorized as pre-FDAAA and 41 (29%) as post-FDAAA. All 142 trials were funded by industry and 105 (74%) were phase III, 33 (23%) phase II, and 4 (3%) phase II/III. In addition, the results of 107 (75%) of the trials were interpreted by the FDA to be positive, 17 (12%) as equivocal, and 18 (13%) as negative (Table 2).

**Table 2:** Characteristics of 142 efficacy trials supporting FDA approval of NDAs for neuropsychiatric conditions, 2005–2014

	No. (%) (*n* = 142)	No. registered (%)	No. results reported (%)	No. published (%)
FDAAA applicability				
Pre-FDAAA	101 (71%)	65 (64%)	10 (10%)	91 (90%)
Post-FDAAA	41 (29%)	41 (100%)	41 (100%)	41 (100%)
Pivotal status				
Pivotal	92 (65%)	78 (85%)	40 (43%)	90 (98%)
Non-pivotal	50 (35%)	28 (56%)	11 (22%)	8 (84%)
Study location				
All USA	65 (46%)	50 (77%)	14 (22%)	61 (94%)
Some USA	54 (38%)	41 (76%)	29 (54%)	48 (89%)
None USA	23 (16%)	15 (65%)	8 (35%)	23 (100%)
Study phase				
Phase II	33 (23%)	17 (52%)	7 (21%)	28 (85%)
Phase III	105 (74%)	87 (83%)	44 (42%)	102 (97%)
Phase II/III	4 (3%)	2 (50%)	0 (0%)	2 (50%)
Randomization				
NA (single-group)	5 (4%)	2 (40%)	0 (0%)	5 (100%)
Randomized	136 (96%)	103 (76%)	51 (37%)	126 (93%)
Non-randomized	1 (1%)	1 (100%)	0 (0%)	1 (100%)
Blinding				
Double-blinded	135 (95%)	102 (76%)	51 (37%)	125 (92%)
Open-label	7 (5%)	4 (57%)	0 (0%)	7 (100%)
Comparators				
Placebo only	94 (66%)	70 (74%)	42 (45%)	86 (91%)
Active comparator only	8 (6%)	5 (63%)	1 (13%)	7 (88%)
Placebo and active comparator	29 (20%)	23 (79%)	6 (21%)	28 (97%)
Lower-dose comparator only	4 (3%)	4 (100%)	2 (50%)	4 (100%)
No comparator	7 (5%)	4 (57%)	0 (0%)	7 (100%)

### Clinical trial registration and results reporting

**Fig. 2:**
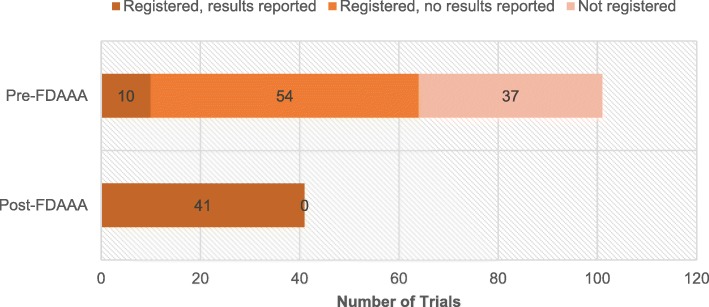
Registration and results reporting status of trials supporting FDA indications by FDAAA applicability, 2005–2014. Post-FDAAA trials were significantly more likely to be registered (100% vs 64%; *p* < 0.001) and report results (100% vs 10%; *p* < 0.001) than pre-FDAAA. Outcomes were compared by two-tailed Fisher exact tests

The FDAAA was followed by significantly greater proportions of trial registration and results reporting. Pre-FDAAA, 64% (65 of 101) of clinical trials were registered on ClinicalTrials.gov, while 100% (41 of 41) of post-FDAAA trials were registered (*p* < 0.001; Fig. 2). Similarly, pre-FDAAA, 10% (10 of 101) of clinical trials reported results on ClinicalTrials.gov, while 100% (41 of 41) of post-FDAAA trials reported results (*p* < 0.001; Fig. 2); the results of 32 of 41 (78%) FDAAA trials were reported within 30 days of drug approval.

### Publication and published interpretations

**Fig. 3:**
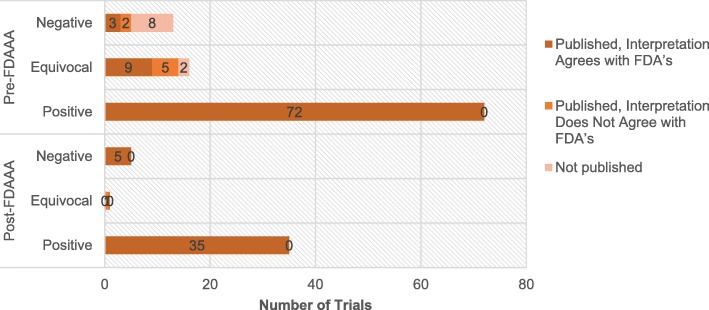
Publication status and publication-FDA agreement of neuropsychiatric Trials by FDAAA applicability and by trial results. Overall, more post-FDAAA trials were published (100% vs 90%; *p* = 0.06) and the publications were in agreement with the FDA’s interpretation (98% vs 93%; *p* = 0.28), but neither outcome was significant. When stratified by results, trials with positive results were all published during both pre-FDAAA (72 of 72) and post-FDAAA (35 of 35). When trials with negative results were examined in isolation, the publication rate was significantly higher after FDAAA as compared to before (5 of 5 vs 5 of 13; *p* = 0.04). There were not enough equivocal trials to draw comparison. All comparisons were based on two-tailed Fisher exact tests

Pre-FDAAA, among 72 positive trials, none were unpublished or published with misleading interpretation (Table 3). In contrast, among 29 non-positive trials, 10 were not published and 7 were published with misleading interpretations (Fig. 3). Post-FDAAA, among 35 positive trials, again none were unpublished and none were published with a misleading interpretation. In addition, among 6 non-positive trials, none were unpublished and 1 was published with a misleading interpretation. The publications of the following new drugs had misleading interpretations: droxidopa (Northera) of Chelsea Therapeutics, dalfampridine (Ampyra) of Acorda Therapeutics, iloperidone (Fanapt) of Vanda Pharmaceuticals, milnacipran hydrochloride (Savella) of Forest Research and Cypress Bioscience, and rulfinamide (Banzel) of Novartis (Additional file 3).

**Table 3:** Publication and publication-FDA agreement of trials supporting FDA approval of NDAs with neuropsychiatric indications with positive, equivocal, and negative results

	Pre-FDAAA			Post-FDAAA		
	FDA interpretation of the trial results, no. (%)			FDA interpretation of the trial results, no. (%)		
	Positive (*n* = 72)	Equivocal (*n* = 16)	Negative (*n* = 13)	Positive (*n* = 35)	Equivocal (*n* = 1)	Negative (*n* = 5)
Published, interpretation agrees with FDA’s	72 (100%)	9 (56%)	3 (23%)	35 (100%)	0 (0%)	5 (100%)
Published, interpretation does not agree with FDA’s	0 (0%)	5 (31%)	2 (13%)	0 (0%)	1 (100%)	0 (0%)
Not published	0 (0%)	2 (13%)	8 (62%)	0 (0%)	0 (0%)	0 (0%)

Trials were classified as positive, negative, or equivocal based on the FDA’s interpretation of the results. Published interpretation of the trial with the FDA’s interpretation for each trial. The two were considered in agreement if the interpretations were both categorized as positive, negative, or equivocal, and no major contradictions existed between the two statements. Negative and equivocal trials were combined into a single group as non-positive trials when calculating publication bias

### Publication bias

Pre-FDAAA, positive trials were more likely to be published (relative risk [RR] = 1.52; 95% confidence interval [CI] = 1.17–1.99; *p* = 0.002) and published without misleading interpretations (RR = 2.47; CI = 1.57–3.73; *p* < 0.001) than those with non-positive results. In contrast, post-FDAAA, positive trials were equally likely to have been published (RR = 1; CI = 1–1, *p* = NA) and published without misleading interpretations (RR = 1.20; CI = 0.84–1.72; *p* = 0.30). The likelihood of publication bias pre-FDAAA vs post-FDAAA was greater for publication of positive vs non-positive trials (RRR = 1.52; CI = 1.16–1.99; *p* = 0.002) and for publication without misleading interpretations (RRR = 2.06, CI = 1.17–3.61, *p* = 0.01).

## Discussion

In this retrospective cohort study of 142 trials supporting the approval of 37 neuropsychiatric therapeutics approved by the FDA between 2005 and 2014, post-FDAAA trials were uniformly registered, reported results, published, and published without misleading interpretations. As compared to pre-FDAAA trials, proportions of trials that were registered and reporting results on ClinicalTrials.gov were significantly higher and the degree of publication bias was lower. Our results suggest that the FDAAA likely contributed to improving the registration, results reporting, and publication of clinical trials supporting FDA approval of new drugs used to treat neuropsychiatric indications, although other factors may also have been in play.

Prior work examining clinical trials supporting FDA approval of new drugs in 2012, many of which were completed before the FDAAA was enacted, found 57% were registered and 20% reported results on ClinicalTrials.gov [5]. Among trials supporting approval of neuropsychiatric drugs, we found similar rates among pre-FDAAA trials, 63% and 10%, but also showed that, among post-FDAAA trials, 100% were registered and reported results. A study involving trials supporting FDA approval of cardiovascular and diabetic drugs showed a similar association between the FDAAA and trial registration and results reporting [35]. Significantly improved clinical trial registration and results reporting after FDAAA enactment might have been anticipated, given the explicit requirement to require trial registration among all trials investigating FDA-regulated products and the alignment between this requirement and the ICMJE’s trial registration policy. Follow-up studies will be needed to determine whether these improvements persist over time. But over the near term, these findings suggest that the FDAAA contributed to improving selective registration and results reporting of clinical trials supporting FDA approvals.

As discussed previously, earlier studies have consistently demonstrated significant publication bias: positive trials are more likely to be published and published accurately or completely than non-positive trials. In our study, such publication bias was observed only among trials that were completed prior to FDAAA enactment, but not afterwards. In addition, overall rates of clinical trial publication were quite high, challenging the assumption that selective publication is worse among clinical trials of neuropsychiatric drugs than for other types of drugs. It is important to note that the “effective publication rate,” as perceived by clinicians and patients, may be lower because publications for some trials were more difficult to locate and were only found using specific trial data in multiple search engines. It is likely that these more-difficult-to-locate publications have a more limited impact on clinical practice, as practicing clinicians and patients have limited time, information, and training to conduct systemic and comprehensive literature searches.

Our findings have important implications for understanding the impact of the FDAAA and for developing future strategies to improve selective publication and outcome reporting more broadly. The FDAAA applies only to trials of medical products regulated by the FDA. But the practice of medicine includes not only the use of medical products to improve patient outcomes, but also behavioral, surgical, and other procedural interventions, as well as health system interventions. To ensure that the medical literature is as unbiased and representative as is possible, rules and regulations like the FDAAA, which mandate registration and results reporting in a publicly accessible database and that apply to all clinical research and health system studies, may be an effective strategy to promote comprehensive registration, results reporting, and publication.

Several factors should be considered in the interpretation of our findings. First, our study is cross-sectional and can only establish associations, not causality. Other reasons beyond the FDAAA, including academic advocacy for clinical trial data transparency, may have accounted for trial sponsors’ and investigators’ decisions to register, report results, and publish the findings of the clinical trials. More studies are needed to disentangle the impact of the FDAAA on clinical trial registration, results reporting, and publication from other factors that may have contributed to these trends, especially the ICMJE clinical trial registration policy—although it is not clear that this policy would affect either results reporting or publication. Second, we limited our search of trial registration to ClinicalTrials.gov. It is possible that trials conducted pre-FDAAA used other registers. Third, for trials determined to be unregistered or unpublished, we did not contact sponsor companies for confirmation. Fourth, our sample is limited to phase II and III trials supporting neuropsychiatric drugs, and the sample size of post-FDAAA trials, at 41, is relatively small. Fifth, our study was focused on pre-marketing phase II and III trials evaluating neuropsychiatric drugs successfully approved by the FDA. Our findings may not be generalized to phase I and phase IV post marketing trials, trials evaluating drugs that were not approved by the FDA, as well as trials for other types of drugs for which the registration and publication have yet to be characterized. Finally, our study was focused on the reporting and publication of trials’ primary results and did not examine reporting or publication of secondary and safety outcomes.

## Conclusions

For clinical trials supporting the FDA approval of new drugs for neuropsychiatric indications, the proportions of trials that were registered and reporting results on ClinicalTrials.gov were significantly higher and publication bias was significantly lower after the passage of the FDAAA in 2007, suggesting that the FDAAA likely contributed to the reduction of selective registration and results reporting and to mitigating publication bias. These findings have important implications for understanding the potential impact of the FDAAA, along with other initiatives that may have improved research reporting, and for developing future strategies to improve selective publication and outcome reporting more broadly.

## Additional files

Additional file 1: Additional information for the Methods section. Details on how the trial results were categorized as positive, equivocal, or negative and on the search strategy for publications. (DOCX 17 kb)

Additional file 2: Characteristics of New Drug Applications approved by the FDA between 2005 and 2014 for the treatment of neurologic and psychiatric conditions (*n* = 37). (DOCX 15 kb)

Additional file 3: List of trials supporting FDA NDA approval with neuropsychiatric indications published with interpretations not in agreement with the FDA’s. For each trial published with interpretations that disagree with those of the FDA, we provided the FDAAA status, trial funding source, FDA interpretation of trial results, quote of the FDA conclusion, publication interpretation, and quote of the published conclusion. (DOCX 35 kb)

## Abbreviations

FDA: Food and Drug Administration
FDAAA: Food and Drug Administration Amendments Act
FDAMA: Food and Drug Administration Modernization Act
ICMJE: International Committee of Medical Journal Editors
NDA: New Drug Application
NIH: National Institutes of Health
