# Supplementary material for: Registration, results reporting, and publication bias of clinical trials supporting FDA approval of neuropsychiatric drugs before and after FDAAA: a retrospective cohort study
Source: Trials. 2018 Oct 23;19:581. doi: 10.1186/s13063-018-2957-0 (PMC6199729; doi:10.1186/s13063-018-2957-0)
Supplement: Supplementary file 1 — Additional information for the Methods section. Details on how the trial results were categorized as positive, equivocal, or negative and on the search strategy for publications. (DOCX 17 kb) [file 13063_2018_2957_MOESM1_ESM.docx]

Additional File 1

### Additional Method

**Classification of Trials results**

Trials were categorized as positive if the primary outcomes achieved statistical significance according to the FDA’s analysis and if the FDA medical reviewer did not provide reasoning to question the positive results. Trials were categorized as negative if the primary outcomes did not achieve statistical significance or if the FDA medical reviewer stated explicitly that the trials were considered “negative”. Trials were categorized as equivocal in the remaining two scenarios: 1) when the FDA medical reviewers questioned statistically significant outcomes for reasons such as the results being highly improbable or flaws in the study design or execution; and 2) when the FDA considered a trial with statistically insignificant primary outcomes as supportive evidence to approve the drug in light of significant secondary outcome(s).

### Determination of Publication Status

We searched PubMed for full-length publications. The following types of publications were excluded: abstracts, conference reports, reviews, meta-analyses, sub-group or post-hoc analyses. Publications reporting multiple trials were also excluded unless the details and results of each trial were reported separately with sufficient details.

One investigator (CXZ) performed the initial search in a step-wise fashion. 1) For registered trials, we first checked the linked publication. 2) We searched in PubMed using the NCT number. 3) We searched in PubMed using a combination of the alternative, generic, or brand names of the study drugs, drug indications, trial IDs, trial acronyms, numbers of participants randomized, comparators, and study time frames. 4) Google Scholar was sometimes employed to help identify the correct PubMed entry. One major limitation of the PubMed search engine is that, as of now, it does not search beyond the titles and abstracts for many publications. However, sometimes the information relevant to match a specific publication with a given trial were mentioned only in the full text, resulting in false negative. For this reason, Google Scholar has the capacity to search among the full texts of various publications hosted on various online platforms and databases, such as ResearchGate in the format of PDF. All publications identified via Google Scholar were able to be located using the exact titles.

Publications are considered a match to the trials identified from the FDA source when the following trial characteristics are the same in both sources: Interventions (including dosing and comparators), indications, trial design (blinding, randomization, trial length), and study populations (total randomized and numbers in each arm). Matched publications for all but 10 pre-FDAAA trials were located. A second investigator (JEB) and a librarian specialized in medical literature each independently performed searches for the 10 trials without matching publications in April 2017 and May 2018 respectively. No additional matching publications were found and those trials were considered unpublished.
