# Supplementary material for: Registration, results reporting, and publication bias of clinical trials supporting FDA approval of neuropsychiatric drugs before and after FDAAA: a retrospective cohort study
Source: Trials. 2018 Oct 23;19:581. doi: 10.1186/s13063-018-2957-0 (PMC6199729; doi:10.1186/s13063-018-2957-0)
Supplement: Supplementary file 2 — Characteristics of New Drug Applications approved by the FDA between 2005 and 2014 for the treatment of neurologic and psychiatric conditions (n = 37). (DOCX 15 kb) [file 13063_2018_2957_MOESM2_ESM.docx]

**Additional File 2**

**Table:** Characteristics of new drug applications approved by the FDA between 2005 and 2014 for the treatment of neurologic and psychiatric conditions (n=37).

|  | **No. (%)** |
| --- | --- |
| **Approval Year** |  |
| 2005-2006 | 4 (11%) |
| 2007-2008 | 9 (24%) |
| 2009-2010 | 10 (27%) |
| 2011-2012 | 6 (16%) |
| 2013-2014 | 8 (22%) |
| **Agent type** |  |
| Pharmacologic | 34 (92%) |
| Biological | 3 (8%) |
| **Orphan status** |  |
| Yes | 9 (24%) |
| No | 28 (76%) |
| **Priority Review Status** |  |
| Yes | 6 (16%) |
| No | 31 (84%) |
| **Approval pathway** |  |
| Accelerated | 1 (3%) |
| Regular | 36 (97%) |
| **Therapeutic Indication** |  |
| Neurological | 23 (62%) |
| Psychiatric | 14 (38%) |
