# Supplementary material for: Registration, results reporting, and publication bias of clinical trials supporting FDA approval of neuropsychiatric drugs before and after FDAAA: a retrospective cohort study
Source: Trials. 2018 Oct 23;19:581. doi: 10.1186/s13063-018-2957-0 (PMC6199729; doi:10.1186/s13063-018-2957-0)
Supplement: Supplementary file 3 — List of trials supporting FDA NDA approval with neuropsychiatric indications published with interpretations not in agreement with the FDA’s. For each trial published with interpretations that disagree with those of the FDA, we provided the FDAAA status, trial funding source, FDA interpretation of trial results, quote of the FDA conclusion, publication interpretation, and quote of the published conclusion. (DOCX 35 kb) [file 13063_2018_2957_MOESM3_ESM.docx]

**Additional File 3**

| **Table:** Clinical Trials Evaluating Neuropsychiatric Drugs Approved by the FDA between 2005 and 2014 that were Published with Misleading Interpretations as Compared to the FDA Interpretations. | | | | | | |
| --- | --- | --- | --- | --- | --- | --- |
| Drug Name: Study ID | FDAAA Status | Trial Sponsor | FDA Interpretation | FDA Conclusions | Publication Interpretation | Published Conclusions |
| Droxidopa (Northera) 301 | Post-FDAAA | Chelsea Therapeutics | Equivocal | "These results were considered to be implausible, and quite remarkable, different from all other data....1) If data from Site 507 were deemed acceptable, that would mean that the dossier included a single positive study. But with only 1 positive study and one site disproportionately responsible for the favorable treatment effect, the data would not constitute sufficient evidence of efficacy upon which to base an approval action. 2) If data from Site 507 were deemed inadmissible, then Study 301 was not positive; none of the studies were positive."^1^ | Positive | "In patients with symptomatic nOH, droxidopa improved symptoms and symptom impact on daily activities, with an associated increase in standing systolic BP, and was generally well tolerated published the analysis per protocol".^2^ |
| Dalfampridine (Ampyra)  202 | Pre-FDAAA | Acorda Therapeutics Inc | Negative | "There were no statistically significant differences between any dose and placebo. Independent analyses by Dr. Joo-Yeon Lee of Pharmacometrics has shown no dose response in the range studied for the percent change from baseline in walking speed". ^3^ | Equivocal | "This phase 2 study suggests that a subgroup of patients, when treated with fampridine, experiences a clinically relevant improvement in walking ability, which is sustained for at least 14 weeks"^4^ |
| Iloperidone (Fanapt) 3000 | Pre-FDAAA | Vanda Pharmaceuticals Inc | Negative | "Thus either approach to defining the sample for this study (whether it's FDA preferred or sponsor preferred) yields a negative result for iloperidone. With the sponsor's preferred analysis including all randomized patients, the superiority of haloperidol (active comparator) over the primary iloperidone (study drug) group (8+12mg) is statistically significant. This study, therefore, provides no support for iloperidone (study drug) but does suggest the statistically significant superiority of haloperidol (active control) over iloperidone (study drug).” FDA reviewer also pointed out that the protocol of 3000 specified that no comparison of individual doses against place can be done unless the combined group 12mg+8mg are shown to be significant, since the combined dose was not significant, then no individual dose comparison can be done and the study is negative.^5^ | Positive | Publication concluded that 3000, 3004 and 3005 are all positive. The positive conclusion for 3000 was partially supported by comparing the outcomes of individual doses against placebo, a practice not supported by the protocol as pointed out the FDA reviewer Publication also did not comment on the fact that the study drug was less effective than the active control. Also in the abstract session is the mentioning of other analysis. "Additional analysis in patients who received active treatment for at least 2 weeks indicated comparable efficacy score reductions at 6 weeks for patients receiving iloperidone 20 to 24 mg/d versus those receiving haloperidol or risperidone". The publication stated, at the end of the abstract that "These trials indicate that iloperidone is effective for the treatment of schizophrenia."^6^ |
| Iloperidone (Fanapt)  3004 | Pre-FDAAA | Vanda Pharmaceuticals Inc | Equivocal | "…....we still do not find this study an acceptable source of evidence. Although the results for both dose groups in the all-randomized patients’ analysis are positive, there is a striking difference in outcomes for the schizophrenic (intended indication) and the schizoaffective (not intended indication) subgroups. The analysis focusing only on the schizophrenic subgroup is not even close to positive for either dose group (p=0.306 for 10-16mg/day and p=0.581 for 4-8mg/day).”   Following this conclusion, the FDA reviewer stated " I think the data for that trial are fatally pathological, and one cannot reasonably pool data from the schizophrenic and schizoaffective subgroups. It is not that I fundamentally object to polling data from schizophrenic and schizoaffective patients (we have accepted this approach many times in the past), but for this study, where the positive findings in the schizoaffective patients is by the sponsor's own admission an 'anomaly,' there is no justification for such a pooling. Thus I find this study uninterpretable.”^5^ | Positive | Same as above. "These trials indicate that iloperidone is effective for the treatment of schizophrenia." ^6^ |
| Iloperidone (Fanapt) 3005 | Pre-FDAAA | Vanda Pharmaceuticals Inc | Equivocal | FDA noted that” the effect size observed in Study 3005 was greater in active control than in both doses of iloperidone”. And that” the observation in study 3005 that the positive effect for iloperidone over placebo was coming almost entirely from the non-US sites.” The reviewer, in summarizing the findings in study 3005, pointed out that stated that the data collected from the US sites for study 3005 were uninterpretable I am persuaded by the sponsor's argument that the lack of efficacy in the US sites for study 3005 should not rule out this study as a source of evidence. As they point out, risperidone also failed in the US sites, and thus, data from these sites is simply uninterpretable.” The conclusion of the FDA was “For study 3005, only the analysis focused on the schizophrenic subgroup shows superiority of iloperidone over placebo. In the sponsor's preferred analysis, iloperidone fails to show superiority to placebo and, at the same time, risperidone appears to be statistically significantly superior to iloperidone.”^5^ | Positive | Same as above, publication concluded that one of the dose in study 3005, 20–24 mg/d [P = 0.010]). "Active controls were also significantly more effective than placebo in each trial, thus validating the trials... These trials indicate that iloperidone is effective for the treatment of schizophrenia." No comparison was drawn between the active control with iloperidone. No statement was made regarding the data from the US study sites were uninterpretable. ^6^ |
| Milnacipran Hydrochloride (Savella)  02 | Pre-FDAAA | Forest Research Institute and Cypress Bioscience | Equivocal | The FDA medical reviewer in more than one places in the review emphasized that this trial fail to demonstrate effect as far as pain is concerned: “analysis of the ‘pain only’ responders does not indicate that there is a significant effect of MLN(Savella) on pain” and the treatment effect is “driven by the patient global response outcome rather than the pain or function outcome, when studied in isolation, statistically significant treatment effects for pain and function were not demonstrated”.   In the risk benefit section of the summary review, the FDA again stated that "an unusual finding in this application is that, while the product appears to be effective when measured according to a prespecified responder definition, the results on the individual components of that responder definition, pain, function and a patient global evaluation, were not consistently statistically significant in the post-hoc analyses performed by the clinical/statistical review team. In particularly, the dominant feature of FM is pain and the results of the team's analyses of the individual pain endpoints did not demonstrate a statistically significant treatment effect for Savella (milnacipran), on the pain endpoint in either of the clinical trials."^7^ | Positive | For trial 02 of milnacipran (Savella), the author of the publication concluded in the abstract that “both doses (100 and 200 mg/d) were associated with significant improvements in pain and other symptoms.”^8^ |
| Milnacipran Hydrochloride (Savella)  031 | Pre-FDAAA | Forest Research Institute and Cypress Bioscience | Equivocal | As in the case for the other milnacipran trials, the FDA reviewer stated that "It is important to note that, in this study, these analyses did demonstrate that the treatment effect for Savella (milnacipran) found in the composite outcomes appears to be driven by the patients’ global response outcomes rather than the pain or function outcome. When studied in isolation, statistically significant treatment effect for pain and function were not demonstrated for Savella compared to placebo.”^7^ | Positive | Author of the publication, stated that the milnacipran achieved statistical significant on the FM pain responders and significant pain reduction was observed after week 1 with both dose and that at "15 weeks, milnacipran 200 mg/day led to significant improvements over placebo in pain (real-time, daily and weekly recall; all measures, p < 0.05)" and the overall conclusion was that “Milnacipran is safe and effective for the treatment of multiple symptoms of FM."^9^ |
| Rufinamide (Banzel) AE/ET1 | Pre-FDAAA | Novartis | Equivocal | FDA conclusion in summary review: “Dr. Siddiqui noted that the sponsor had included the placebo group in its dose response analyses. Further, he noted that the dose groups were coded inappropriately (non-proportional to the actual dose). When the doses were coded proportionally to the actual dose in his dose-response analysis, and placebo was excluded, the p-value for the dose response slope was 0.086, implying none of the dose differed materially.”^10^ | Positive | The author concluded in the abstract of the publication that “in the linear trend of dose response for seizure frequency per 28 days in the double-blind treatment phase was statistically significant in favor of rufinamide (p=0.003).^11^ |

**Reference List**

1. *Summary Review Application Number 203202Orig1s000 Center for Drug Research and Evaluation. Food and Drug Administration. Https://Www.Accessdata.Fda.Gov/Drugsatfda_Docs/Nda/2014/203202Orig1s000SumR.Pdf. Published February 18, 2014. Accessed August 7, 2017*.

2. Kaufmann H, Freeman R, Biaggioni I, et al. Droxidopa for neurogenic orthostatic hypotension: a randomized, placebo-controlled, phase 3 trial. *Neurology*. 2014;83(4):328-335.

3. *Summary Review. Application Number 22-250s000. Center for Drug Research and Evaluation. Food and Drug Administration. Https://Www.Accessdata.Fda.Gov/Drugsatfda_Docs/Nda/2010/022250s000_SumR.Pdf. Published January 18, 2010. Accessed August 7, 2017*.

4. Goodman AD, Brown TR, Cohen JA, et al. Dose comparison trial of sustained-release fampridine in multiple sclerosis. *Neurology*. 2008;71(15):1134-1141.

5. Summary Review. Application Number 22-192. Center for Drug Research and Evaluation. Food and Drug Evaluation https://www.accessdata.fda.gov/drugsatfda_docs/nda/2009/022192s000_SumR.pdf. Published March 27, 2009. Accessed August 7, 2017.

6. Potkin SG, Litman RE, Torres R, Wolfgang CD. Efficacy of iloperidone in the treatment of schizophrenia: initial phase 3 studies. *Journal of Clinical Psychopharmacology*. 2008;28(2):S4-S11.

7. Summary Review for Regulatory Action for Application Number 22-256. FDA Center for Drug Evaluation and Research Division of Anesthesia, Analgesia, and Rheumatology Products. October 2008:11. https://www.accessdata.fda.gov/drugsatfda_docs/summary_review/2009/022256s000SumR.pdf. Published January 13, 2009. Accessed August 7, 2017.

8. Clauw DJ, Mease P, Palmer RH, Gendreau RM, Wang Y. Milnacipran for the treatment of fibromyalgia in adults: A 15-week, multicenter, randomized, double-blind, placebo-controlled, multiple-dose clinical trial. *Clinical Therapeutics*. 2008;30(11):1988-2004.

9. Mease PJ, Clauw DJ, Gendreau RM, et al. The Efficacy and Safety of Milnacipran for Treatment of Fibromyalgia. A Randomized, Double-blind, Placebo-controlled Trial. *The Journal of Rheumatology*. 2009;36(2):398-409.

10. *Summary Review. Application Number 21-911. Center for Drug Research and Evaluation. Food and Drug Administration. Https://Www.Accessdata.Fda.Gov/Drugsatfda_Docs/Nda/2008/021911s000_SumR.Pdf. Published November 6, 2008. Accessed August 7, 2017*.

11. Elger CE, Stefan H, Mann A, Narurkar M, Sun Y, Perdomo C. A 24-week multicenter, randomized, double-blind, parallel-group, dose-ranging study of rufinamide in adults and adolescents with inadequately controlled partial seizures. *Epilepsy Research*. 2010;88(2-3):255-263.
